# Utilization of contraceptives by persons living with HIV in Eastern Uganda: a cross sectional study

**DOI:** 10.1186/s12978-015-0030-y

**Published:** 2015-05-07

**Authors:** Richard Ekorinyang

**Affiliations:** World Vision Uganda, P.O. Box 5319, Kampala, Uganda

**Keywords:** Contraceptives, Utilization, HIV, Eastern Uganda

## Abstract

**Background:**

In Uganda, there has been an increase in use of contraceptives by 6% from 2006 to 2011 among married women. During the same period HIV prevalence had gone up by 0.9%. Lack of use of contraceptives especially among persons living with HIV may escalate the spread of the virus. The purpose of the study was to determine the rate of contraceptive use and associated factors among persons receiving HIV care and treatment in Eastern Uganda.

**Methods:**

A cross-sectional study was conducted in 4 public hospitals of Mbale, Kapchorwa, Atutur and Pallisa in Eastern Uganda. In total, 300 respondents comprising of women aged (15–49) and men (15–54) years were interviewed using interviewer administered questionnaires. However, data from 298 respondents were analyzed using multinomial logistic regression at α = 0.05 in STATA statistical software (Version 10).

**Results:**

Approximately 62% (185/298) of persons living with HIV had used contraceptives within the three months preceding the study. Among the significant predictors, higher proportions of female respondents aged 36–49 years used injectables and male aged 50–54 years used condoms (p = 0.030 and p = 0.034, respectively). Furthermore, higher proportions of respondents with primary, secondary and tertiary education levels were more likely to use condoms (p = 0.004, p = 0.000 and p = 0.005, respectively) compared with those who never went to school. Besides, condoms were being used by Protestants (p = 0.000) compared to Catholics and Muslims. Also, more female respondents (p = 0.000) used condoms with their partners compared with the male counterparts. The main barrier to contraceptive use among non-users was desire for more children.

**Conclusion:**

More efforts are needed to sensitize and provide contraceptives targeting the illiterate clients, youth, men and believers from different religious sects to increase utilization.

## Introduction

There are great benefits when persons living with HIV use contraceptives. Among the benefits noted; contraceptives prevent unintended pregnancy and are largely used in prevention of mother-to-child transmission [1,2]. Additionally, condoms in HIV care not only serve as contraceptives but also prevent sexually transmitted infections including HIV cross and re-infection [3,4].

In Sub-Saharan Africa, use of modern contraceptives by HIV-positive women has led to a decrease in HIV-positive births by 31% [5]. In comparison, use of contraceptives prevents HIV-positive births (19.7%) better than antiretroviral therapy (ARV)-based PMTCT (8.1%) in Uganda [6]. Despite the fact that contraceptive use among married women was 24.0% in 2006 [7], the national survey in 2011 indicated that it had increased to 30.0% [8]. A similar trend had been noted in Eastern Uganda in which case contraceptive use among married women had increased from 20.0% in 2006 [9] to 26.1% in 2011 [8]. Although services to maximize contraception have been included in all phases of HIV care and treatment in Uganda [10], HIV prevalence rate has gone up from 6.4% in 2006 to 7.3% in 2011 among persons aged 15 to 49 years [11]. The rise in new HIV infections is partly attributed to inconsistent use of condoms [12]. The factors influencing people’s decisions on contraceptive use in Sub-Saharan Africa are age, education and marital status, among others [13].

Contraceptive use among persons living with HIV in Kabale district, Western Uganda is 55.1% [14]. However, no knowledge on contraceptive use among persons living with the virus in other parts of the country was available at the time of this study. I, therefore, undertook this study to establish the rate of contraceptive use by persons living with HIV in Eastern Uganda, taking into account the type of contraceptives and the factors influencing their use in relation to age, sex, religion, education, marital status and whether or not they were using ARVs. The reasons for not using contraceptives among some of the clients were also documented.

## Methods

A cross-sectional study was conducted in four public hospitals of Mbale, Kapchorwa, Atutur and Pallisa in Eastern Uganda. A list of all public hospitals under the Eastern Uganda cluster was made [7]. Mbale hospital, one of the two regional referral hospitals in Eastern Uganda, was purposively selected because it is more centrally located and provides technical support to most of the public hospitals in the study area than the other. The other 3 general hospitals were selected by simple random procedure. In other words names of the 6 hospitals were written on pieces of paper, dropped in a box and picking done by replacement until 3 were picked.

The sample size of the interviewees was determined using Kish and Leslie formula;

$$ \mathrm{n}=\frac{{\mathrm{Z}}^2\mathrm{P}\left(1-\mathrm{P}\right)}{{\mathrm{d}}^2} $$

n = the minimum sample size required; Z = alpha risk expressed in Z-score = 1.96 at 95% confidence limit,

d = accepted margin of error at 95% confidence limit = +/−5%

P = expected proportion of clients utilizing contraceptives.

Considering that the national contraceptive prevalence was 24% [7]; the calculated minimum sample size of interviewees was 280 and was adjusted to 300.

The interviewees were proportionately distributed among the four hospitals based on the number of active HIV clients (HIV positive clients who had come to the HIV clinic at least once within the previous three months of follow up). The figures used were from hospital-based Health Management Information System (HMIS) data reported in the 3rd quarter (January to March of 2011). Given that the active clients who accessed services in the hospitals were 6451; Atutur 1677, Pallisa 1099, Kapchorwa 1063 and Mbale 2612, the proportionate sample size for the HIV positive clients was; Atutur 78; Pallisa 51; Kapchorwa 49 and Mbale 122.

Out of the 300 persons who were picked from the hospital HIV clinics, 87 men aged 15 to 54 years and 213 women aged 15 to 49 years were interviewed. HIV positive clients who had reported for their follow up were told about the study, invited to participate and only those who consented to the interview were allowed to participate. Having determined the number of interviewees per hospital, whoever came to the HIV clinic for their follow up visits and met the selection criteria during the period of data collection was interviewed. The disparity in sex distribution was due to the interview process which had no proportionate distribution at this point. However, the difference in the age limits of men and women was considered as the reproductive age range for the men and women accordingly. Categories of age groups were broken into 15 to18 years, 19 to 25 years, 26 to 35 years, 36 to 49 years and 50 to 54 years. These age intervals in the design of the study were selected purposively considering adolescents, early adulthood, middle and late adulthood during reproductive age. The study excluded expectant HIV positive women because they were not expected to be using contraceptive methods used outside pregnancy other than condoms at the time. Adolescents aged below 15, women above 49 and men above 54 were equally excluded with the expectation that they would not be sexually active, hence no need for contraception.

A structured interviewer administered questionnaire was used for collecting data. Three certificate nurses (data collectors) were trained on the data collection tool, selection of participants, data quality and ethical considerations. Throughout the data collection the principal investigator was available for any support.

Data were coded and entered into statistical package for social scientists (SPSS version 16.0) and cleaned. Exploration of data was by Microsoft office excel professional 2007. Frequencies and percentages were generated. Data were analyzed using a multinomial logistic regression model at α = 0.05 in STATA statistical software (Version 10). Any factor with a p-value of less than 0.05 at 95% confidence interval was considered statistically significant. Because only 2 respondents were obtained for the age group 15 to 18 years, it was not considered in the statistical analysis.

The study received approval from the Faculty of Health Sciences, Ugandan Martyrs University and Mbale Regional Referral Hospital research Review Board. Written consent was sought from all the hospitals where the study was conducted for back up. All interviews were conducted in an area that allowed confidentiality after obtaining the respondents’ written consent.

## Results

### Socioeconomic and demographic characteristics of respondents

About half of the respondents 50.3% (150/298) were aged between 36 and 49 years (Table 1). Female respondents were 70.8% (211/298); and those who were married were 68.8% (205/298). By religious affiliation, most of the respondents 44.3% (131/298) were protestant. A majority of the respondents 63.4% (189/298) attained primary education and 11.1% (33/298) did not have any formal education.

**Table 1:** **Socio-demographic characteristics of persons living with HIV in Eastern Uganda, 2011 (N = 298)**

**Demographic characteristics**		**Frequency**	**Percentage**
Sex	Male	87	29.2
	Female	211	70.8
Age (Years)	19-25	19	6.4
	26-35	105	35.2
	36-49	150	50.3
	50-54	24	8.1
Religion	Catholic	88	29.7
	Protestant	131	44.3
	Muslim	37	12.5
	Pentecostal	40	13.5
Marital status	Married	205	68.8
	Single	14	4.7
	Cohabiting	29	9.7
	Widowed/separated/ divorced	50	16.8
Education level	No formal education	33	11.1
	Primary	189	63.4
	Secondary	59	19.8
	Tertiary	17	5.7

### Contraceptive use

**Figure 1:**
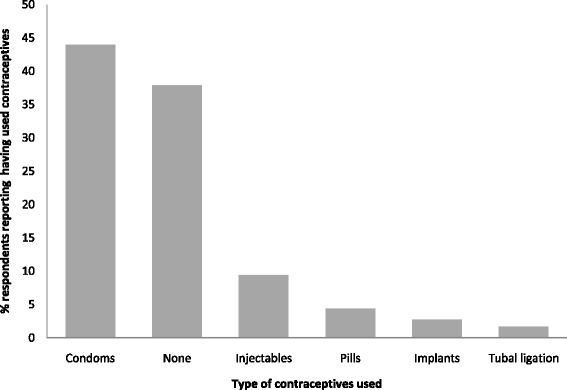
Contraceptive methods used by persons living with HIV in Eastern Uganda, 2011 (N = 298).

The rate of contraceptive use among the respondents was 62.1% (185/298). Condoms were the most popular type of contraceptives used by 44.0% (131/298) of the respondents (Figure 1).

A higher proportion of male respondents aged 50 to 54 years were more likely to use condoms compared with other respondents aged 19 to 25 years and this was found to be statistically significant (p = 0.034) (Table 2). However, there were no significant differences in condom use by respondents in age groups 26 to 35 years and 36 to 49 years compared with those aged 19 to 25 years. Generally, a higher proportion of female respondents were more likely to use condoms with their partners compared with the male respondents and this was found to be statistically significant (p = 0.000). In this study it was found that religion influenced condom use among respondents with fewer Muslims (p = 0.049) using condoms than Catholics, while there was no difference in condom use among respondents from other religions compared to Catholic. Furthermore, higher proportions of respondents with primary, secondary and tertiary education levels were more likely to use condoms (p = 0.004, p = 0.000 and p = 0.005 for the three education levels, respectively) compared with those who never went to school. The study results also revealed that a lower proportion of separated persons (p = 0.000) used condoms than those who were married; while there were no significant differences in proportions among singles and cohabiting persons using condoms compared with those who were married. There was no significant difference found in condom use among those who were on ARVs compared with those who were not.

**Table 2:** **Distribution of contraceptives used by persons living with HIV in Eastern Uganda with respect to selected characteristics, 2011 (N = 296)**

**Independent variables**		**Dependent variables (%)**				
		**Condoms**	**Pills**	**Injectables**	**Implants**	**Tubal ligation**
Age (Years)	19-25	4.6	15.4	17.9	0.0	0.0
	26-35	32.8	38.5	53.6	62.5***	60.0***
	36-49	52.7	46.2	28.6*	37.5	40.0
	50-54	9.9*	0.0	0.0	0.0	0.0
Sex	Male	47.3	0.0	0.0	0.0	0.0
	Female	52.7***	100.0	100.0	100.0	100.0
Religion	Catholic	28.2	23.1	14.3	37.5	0.0
	Protestant	41.2	53.9	57.1	50.0	80.0***
	Muslim	14.5*	15.4	17.9	0.0	0.0
	Pentecostal	16.0	7.7	10.7	12.5	20.0
Education	None	3.8	0.0	14.3	37.5	0.0
	Primary	62.6**	61.5***	57.1	50.0	100.0
	Secondary	25.2***	23.1***	25.0	12.5	0.0
	Tertiary	8.4**	15.4	3.6	0.0	0.0
Marital status	Married	81.7	76.9	60.7	100.0	60.0
	Single	3.8	0.0	7.1	0.0	0.0
	Cohabiting	9.9	15.4	21.4	0.0	0.0
	Separated^x^	4.6***	7.7	10.7	0.0	40.0
ARV use	Yes	82.4	84.6	67.9	37.5	80.0
	No	17.6	15.4	32.1	62.5	20.0

mlogit model: Contraceptives _~_ i.Age i.Sex i.Religion i.Education i.Marital status i.ARV use, (base outcome = Non contraceptive use); Number of observations = 296; Pseudo R^2^ = 0.2529; one, two and three asterisks indicate significant differences from the reference first category at p ≤ 0.05, p ≤ 0.01 and p ≤ 0.001, respectively; ^x^ = Widowed or divorced or separated due to disagreement with spouse.

A higher proportion of respondents who attained primary and secondary education were more likely to use pills than those who never went to school and this was found to be significant (p = 0.000 for both education levels). However, there was no significant difference in use of pills by respondents who attained tertiary education compared with those who never went to school. Age, sex, religion, marital status and use of ARVs had no significant effect on use of pills.

Study results indicated that a higher proportion of respondents aged 36 to 49 years were more likely to use injectables than those aged 19 to 25 and this was found to be statistically significant (p = 0.030). However, there were no significant differences in the use of injectables among other age groups compared with those aged 19 to 25 years. Similarly, a higher proportion of respondents in the age group 26 to 35 years were more likely to use implants compared with those aged 19 to 25 years. Results showed that there was no significant difference in use of implants among other age groups compared with those aged 19 to 25 years. Sex, religion, education, marital status and use of ARVs had no significant effect on the use of injectables and implants.

A higher proportion of female respondents aged 26 to 35 years had undergone tubal ligation compared with those aged 19 to 25 and this was found to be statistically significant (P = 0.000). There was no significant difference found among female respondents aged 36 to 49 years who had undergone tubal ligation compared with those aged 19 to 25 years. Among the respondents who had undergone tubal ligation, protestants were more likely to have done it compared with Catholics with a significant value of (P = 0.000). There were no significant differences in proportions of Muslims and Pentecostals who had undergone tubal ligation compared with Catholics. Sex, education, marital status and use of ARVs had no significant effect on tubal ligation as a means of contraception.

### Why some persons living with HIV did not use contraceptives

**Figure 2:**
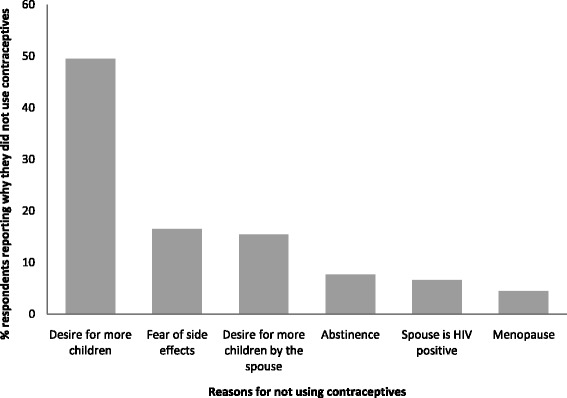
Distribution of factors influencing contraceptive use among persons living with HIV in Eastern Uganda, 2011 (N = 113).

Among the respondents who did not use contraceptives, about half 49.5% (56/113) attributed this to their desire for more children (Figure 2).

A higher proportion of respondents aged 26 to 35 and 36 to 49 years were not likely to use contraceptives for fear of side effects compared with those aged 19 to 25 years and this was found to be statistically significant (p = 0.000 for both) (Table 3). However, there was no significant difference in proportions of male respondents aged 50 to 54 years who were not likely to use contraceptives for fear of side effects when compared with other respondents aged 19 to 25 years.

**Table 3:** **Distribution of factors influencing use of contraceptives by persons living with HIV in Eastern Uganda with respect to selected characteristics, 2011 (N = 90)**

**Independent variables**		**Reasons for not using contraceptives (%)**				
		**Fear of side effects**	**Abstinence**	**Spouse wants more children**	**Spouse is HIV positive**	**Menopause**
Age	19-25	0.0	0.0	21.4	0.0	0.0
	26-35	26.7***	0.0	42.9	33.3	0.0
	36-49	66.7***	57.1	35.7	50.0	100.0
	50-54	6.7	42.9	0.0	16.7	0.0
Sex	Male	6.7	42.9	7.1	0.0	0.0
	Female	93.3	57.1	92.9	100.0	100.0
Religion	Catholic	40.0	42.9	35.7	16.7	50.0
	Protestant	40.0	14.3	35.7	50.0	50.0
	Muslim	13.3	28.6	21.4	16.7	0.0
	Pentecostal	6.7	14.3	7.1	16.7	0.0
Education	None	20.0	0.0	7.1	16.7	0
	Primary	66.7	71.4***	57.1	83.3	100.0
	Secondary	13.3	28.6	28.6	0.0	0.0
	Tertiary	0.0	0.0	7.1	0.0	0.0
Marital status	Married	66.7	71.4	71.4	100.0	75.0
	Single	0.0	0.0	0.0	0.0	0.0
	Cohabiting	33.3	14.3	21.4	0.0	0.0
	Separated^x^	0.0	14.3	7.1	0.0	25.0
Use of ARVs	Yes	53.3	100.0	57.1	83.3	100.0
	No	46.7	0.0	42.9	16.7	0.0

mlogit Reasons for not using contraceptives _~_ i.Age i.Sex i.Religion i.Education i.Marital status i.ARV use (base outcome = desire for children); Number of observations = 90; Pseudo R^2^ = 0.3959; three asterisks indicate significant differences from the reference first category at p ≤ 0.001; ^x^ = Widowed or divorced or separated due to disagreement with spouse.

In this study, a higher proportion of respondents who attained primary education were not likely to use contraceptives compared with those who never went to school because they were abstaining, and this was found to be statistically significant (p = 0.000) (Table 3). Nevertheless, there were no significant differences in proportions of respondents who attained secondary and tertiary education compared with those who never went to school indicating abstinence as a reason for not using contraceptives. On the other hand, respondents’ sex, religion, education, marital status and use of ARVs had no significant effect on fear of side effects as a reason for not using contraceptives. Apart from sex and education, age and other factors investigated had no significant effect on abstinence as a reason for not using contraceptives. Furthermore, age, sex, religion, education, marital status and use of ARVs had no significant effect on desire of more children by the spouse, positive HIV status of the spouse and menopause as reasons for not using contraceptives.

## Discussion

The results show that the rate of contraceptive use is 62% (185/298) among persons living with HIV in Eastern Uganda in comparison to that reported in the western part of the country which was 55.1% [14]. Although the study results for this population were more than average, different studies indicate that the rate of contraceptive use among women nationally was much lower [7-9]. Currently, there has been scale up of inclusion of contraceptive services in HIV care across the Ugandan health system, hence improved access, though primarily for prevention of HIV spread [10,15]. Unlike for the general population, other studies suggest that targeted sensitization about benefits of using contraceptives during client follow-up visits in HIV care facilities could explain the higher rate of contraceptive use [16,17]. These findings seem to suggest that clinicians could explore integrating provision of contraceptives in all levels of service provision to increase access for not only HIV clients but also other patient categories. This would then pose another challenge for policy makers to have medical training institutions incorporate family planning at large in the curricula.

The use of condoms by HIV positive males aged 50 to 54 years; injectables by females aged 36 to 49 years; implants and tubal ligation by females aged 26 to 35 years was higher than for those aged 19 to 25 years. Although it is not clear why a specific contraceptive is preferred by a given age group, it is evident that older people use contraceptives than those in the age group 19 to 25 years. These results are consistent with those from other studies that suggest that the older the people grow, the more they consider using contraceptives probably because they have attained the desired number of children and would love to prevent HIV transmission [18,19].

Although this study revealed that more women used condoms with their partners compared to men, another study indicated the contrary [20]. Meanwhile, the study results are consistent with other studies showing that women readily take up condom use as long as their spouses allow [21,22]. However, separated couples were less likely to use condoms compared with those who were married, possibly because they were less likely to engage in regular sexual activity compared with the latter [23].

Fewer Muslims living with HIV used condoms than the Catholic counterparts, although it is the Catholic faith that opposes artificial contraceptive use [24]. The study however shows that more Protestants underwent tubal ligation than Catholics, probably due to their liberality on contraceptive use compared to the Catholics. This suggests that despite people’s religious beliefs, they may prioritize contraceptive use as long as they understand the benefits such as preventing unwanted pregnancies and sexually transmitted diseases [4].

This study also showed that more persons living with HIV, with primary, secondary and tertiary education used condoms than their counterparts who never went to school. It was also indicated that more of those with primary and secondary education used pills than their counterparts who never went to school. The study findings are consistent with other reports stating literacy as a positive attribute to contraceptive use [25,26]. Despite the fact that universal primary and secondary education programs exist in Uganda, policy makers could explore introduction of adult education programs to increase literacy levels for all.

Another limiting factor to use of contraceptives found in this study was desire for more children. This finding is consistent with other reports that desire for other children is one most common predictor for not using contraceptives [25,27]. In this study, more persons aged 26 to 35 and 36 to 49 years stated fear of side effects as a barrier for contraceptive use compared with those aged 19 to 25 years. Although the study did not seek to inquire about earlier exposure to contraceptives especially hormonal contraceptives among non users, it is possible that some persons in these age groups could have discontinued themselves due to side effects. Discontinuation rates have been implicated for mostly injectables and pills due to side effects, and yet side effects themselves are barriers to use of other contraceptives [19,28].

This study has revealed that more persons who attain primary education abstain from sexual activity to prevent conception compared with those who never went to school. Since abstinence is also a contraceptive method, it is possible that the persons who attained primary education were more aware of the benefits of contraceptive use than their counterparts who never went to school [25,26,29].

### Strengths and weaknesses

This study had the strength of including men rather than having women alone as respondents like many studies do [12,13,21,23,27,28]. However, there were some limitations noted in this study. There was no statistical backing in selection of respondents in reference to men and women creating a potential bias. This applies to the age intervals and range that were also different among the age groups during analysis. It was therefore difficult to generalize results in relation to some other studies. Researchers could explore conducting a similar study having statistically determined proportions of men and women.

## Conclusion

Overall, this study shows that the rate of contraceptive use among persons living with HIV in Eastern Uganda is higher than that in the Ugandan general population. This could be due to accessibility of contraceptives within HIV care facilities. Integration of HIV care and provision of contraceptives facilitates increased uptake of contraceptives among users. Age, literacy, religion and desire for more children were key determinants to use of contraceptives among persons living with HIV. Contraceptive messages targeting the illiterate, youth, men and believers from different denominations need to be designed since these populations varied in use of contraceptives.
